# Motor Imagery during Action Observation: A Brief Review of Evidence, Theory and Future Research Opportunities

**DOI:** 10.3389/fnins.2016.00514

**Published:** 2016-11-21

**Authors:** Daniel L. Eaves, Martin Riach, Paul S. Holmes, David J. Wright

**Affiliations:** ^1^Sport and Exercise Science Section, Teesside UniversityMiddlesbrough, UK; ^2^Research Centre for Health, Exercise and Active Living, Manchester Metropolitan UniversityCrewe, UK

**Keywords:** combined action observation and motor imagery, AO+MI, motor simulation, motor learning, motor rehabilitation, mental practice, observational learning, movement demonstrations

## Abstract

Motor imagery (MI) and action observation (AO) have traditionally been viewed as two separate techniques, which can both be used alongside physical practice to enhance motor learning and rehabilitation. Their independent use has largely been shown to be effective, and there is clear evidence that the two processes can elicit similar activity in the motor system. Building on these well-established findings, research has now turned to investigate the effects of their combined use. In this article, we first review the available neurophysiological and behavioral evidence for the effects of combined action observation and motor imagery (AO+MI) on motor processes. We next describe a conceptual framework for their combined use, and then discuss several areas for future research into AO+MI processes. In this review, we advocate a more integrated approach to AO+MI techniques than has previously been adopted by movement scientists and practitioners alike. We hope that this early review of an emergent body of research, along with a related set of research questions, can inspire new work in this area. We are optimistic that future research will further confirm if, how, and when this combined approach to AO+MI can be more effective in motor learning and rehabilitation settings, relative to the more traditional application of MI or AO independently.

## Introduction

Motor imagery (MI) and action observation (AO) can be regarded as two forms of *motor simulation*, which activate the motor system in the absence of motor execution (Jeannerod, 2001, 2006). MI is a type of mental practice involving the internal generation of visual and kinesthetic aspects of movement, and a large body of research has recommended that practitioners working in motor learning and rehabilitation settings should use MI to improve motor abilities (see Schuster et al., 2011). This can either be as an accompaniment to physical practice to improve behavioral outcomes (e.g., Rozand et al., 2014; Di Rienzo et al., 2015; Ingram et al., 2016), or as a replacement when movement is restricted due to either neurological impairment or injury (e.g., Szameitat et al., 2012; Hoyek et al., 2014; Mateo et al., 2015). It is also well-documented that AO evokes an internal motor representation of the observed movement (also termed “motor resonance”; see Rizzolatti and Sinigaglia, 2010). Consequently, AO has been recommended as a treatment in neurorehabilitation (Buccino, 2014). It also remains a popular and effective tool for enhancing motor learning (see Ste-Marie et al., 2012).

In terms of the associated neural substrates, MI and AO involve motor and motor-related brain areas, which overlap extensively both with one another, and with the regions involved in motor execution (see Grèzes and Decety, 2001; Caspers et al., 2010; Hétu et al., 2013). Although distinct brain structures are identifiable for AO, MI and execution individually (Filimon et al., 2007, 2015; Munzert et al., 2008; Lorey et al., 2013), the case for using MI and AO in motor learning and rehabilitation has been largely predicated on the degree of neural overlap shared with motor execution. It is important to note, however, that while the majority of evidence supports the effectiveness of MI and AO as independent instruction techniques, there is evidence to the contrary (see Braun et al., 2013; Gatti et al., 2013; Sarasso et al., 2015). Furthermore, it is difficult to draw clear conclusions on the mixed results provided across studies that have compared the potential advantages of **motor imagery vs. action observation**, both on motor function and neural processes (e.g., Porro et al., 2007; Filimon et al., 2007, 2015; Szameitat et al., 2012; Gatti et al., 2013; Gonzalez-Rosa et al., 2015; Helm et al., 2015).

KEY CONCEPT 1 **Motor imagery vs. action observation**

Motor imagery (MI) and action observation (AO) have traditionally been considered as separate interventions for improving motor learning and rehabilitation. Recent research is now focusing more on their combined application (i.e., AO+MI), rather than their independent use.

While the vast majority of previous literature has focused on MI or AO in isolation, or on the similarities versus differences between these two forms of motor simulation, there is now an emerging body of research showing the potential advantages for instructing MI *during* AO (i.e., AO+MI; see Vogt et al., 2013). This instruction typically entails imagining the physiological sensations and kinesthetic experiences of action, and synchronizing this motor simulation with the congruent observed action. Importantly, this procedure seems to be relatively easy for healthy adults to follow and, intuitively, offers a closer representational match to the physical action than simulation through either MI or AO alone.

In this article, we discuss the implications of this new research focus, the evidence generated to date and the questions these data pose to theorists, cognitive neuroscientists, and practitioners in sport, exercise and movement rehabilitation. We give particular attention to the evidence published since the influential review by Vogt et al. (2013). First, we briefly review the neurophysiological experiments providing evidence of enhanced motor-cortical activity for AO+MI, compared to either MI or AO alone. We then examine the limited body of research investigating AO+MI effects on motor behavior. This is followed by a discussion regarding the implications of these data for a conceptual framework of *dual-action simulation*, recently proposed by Eaves et al. (2014, 2016) and Vogt et al. (2013). In the final sections, we discuss potential avenues for future research to investigate particular AO+MI delivery methods for specific populations.

## The effects of motor imagery during action observation: empirical evidence

### Neurophysiological evidence

Observing while imagining the same action (i.e., AO+MI) has, up until recently, received relatively little research attention. To date, an emerging body of multimodal neurophysiological work has shown that **cortico-motor activity is significantly increased during AO+MI** compared to when the same action is either observed or imagined individually.

KEY CONCEPT 2 **Cortico-motor activity is significantly increased during AO+MI**

Combined AO+MI produces increased activity in motor-related brain areas, compared to MI or AO alone. There is some evidence that this increased activity during AO+MI is greater than that which would be obtained by simply summing the activity found during independent MI and AO.

Using functional magnetic resonance imaging (fMRI), Macuga and Frey (2012) were among the first to show that the brain regions involved in AO are largely a subset of those involved during combined AO+MI, which in turn are a subset of those involved in AO with synchronized execution. Taube et al. (2015) also reported that AO, MI and AO+MI each have a unique neural signature, involving greater neural activity for AO+MI in the caudal supplementary motor area (SMA), basal ganglia, and cerebellum compared to AO; and bilateral cerebellum, and precuneous compared to MI. Activity in areas such as the SMA and left precentral gyrus was increased during MI compared to AO, while combined AO+MI further increased activity in those regions beyond both MI and AO independently. In two other studies, AO+MI increased the neural activity over and above AO in parts of the cerebellum, inferior frontal gyrus, inferior parietal cortex, SMA (Nedelko et al., 2012), ventral premotor cortex and left insula (Villiger et al., 2013).

Research using multi-channel electroencephalographic (EEG) recordings has also demonstrated differences in cortical activity between AO+MI and the two constituent (i.e., single-action simulation) processes. Stronger event-related desynchronization (ERD; i.e., a decrease in spectral power, associated with event-related cortical activity) was found over the primary sensorimotor areas within the theta, alpha and beta frequency bands during AO+MI compared to AO (Berends et al., 2013), and in lower alpha and beta bands during AO+MI compared to MI (Neuper et al., 2009). More conclusively, Eaves et al. (2016) reported more pronounced electrophysiological activity over primary sensorimotor and parietal regions in the mu/alpha and beta frequency bands for AO+MI, relative to *both* MI and AO in isolation, using a within-subjects design.

Finally, research into observation and imagery effects using single-pulse transcranial magnetic stimulation (TMS) over the motor cortex has produced two particularly important and relevant findings. First, corticospinal excitability, measured through the amplitudes of motor evoked potentials, during both AO and MI of hand gestures is reliably higher than control conditions (e.g., Clark et al., 2004; Williams et al., 2012; see Naish et al., 2014; Grosprêtre et al., 2016 for reviews). Second, AO+MI produces significantly greater facilitation of corticospinal excitability compared to AO (Ohno et al., 2011; Wright et al., 2014, 2016) and, in some cases, MI as well (Sakamoto et al., 2009; Tsukazaki et al., 2012; Mouthon et al., 2015). These effects have been demonstrated across a variety of tasks, including simple and sequential finger movements (Wright et al., 2014, 2016), gross and fine motor tasks (Sakamoto et al., 2009; Ohno et al., 2011) and coordination tasks (Tsukazaki et al., 2012; Mouthon et al., 2015).

In summary, there is now clear evidence for increased and more widespread activity in the motor execution network during AO+MI, relative to observing or imaging actions independently. In some cases, this increased neurophysiological activity during AO+MI has been shown to be greater than the sum of that reported during independent AO and independent MI (e.g., Sakamoto et al., 2009; Taube et al., 2015). As such, the authors of the experiments reviewed in this section have typically recommended AO+MI as the more effective method for motor learning and rehabilitation, compared to either MI or AO alone. At this point, however, there is limited behavioral and clinical evidence to support this claim.

### Behavioral evidence

Using AO+MI to improve motor learning is not a particularly new concept, although interest in this area has substantially increased following the neuroscientific findings discussed above and recent advancements in video technology. Some of the first behavioral studies were conducted in the sport domain, in which AO+MI (then referred to as “video-guided imagery”) improved performance in both a golf putting task (Smith and Holmes, 2004) and a bicep curl strength test (Wright and Smith, 2009) over 6-week long interventions. These improvements were significantly greater than those following MI alone. It therefore appears that **AO+MI may offer an effective adjunct to physical practice**. The initial explanation for these benefits in motor performance was that the visual stimulus (AO) removed the necessity for the participants to generate a visual mental image (Holmes and Calmels, 2008). This would free up attentional space, allowing participants to focus specifically on imagining the kinesthetic aspects of the movement, while the video also provided visual, auditory and temporal cues for successful performance (Smith and Holmes, 2004).

KEY CONCEPT 3 **AO+MI may offer an effective adjunct to physical practice**

Researchers have suggested that AO+MI interventions may be more effective for motor learning than independent MI or AO. The body of evidence to support this claim is small, but the findings are encouraging.

In two recent intervention studies the pattern of results is arguably less clear. Taube et al. (2014) showed a significant reduction in postural sway over a 4-week balance training intervention, in which healthy participants used either MI *or* AO+MI. This reduction, however, was only numerically (i.e., not significantly) larger for AO+MI compared to MI, while there were also no changes in spinal excitability following the training in either group. Sun et al. (2016) also employed a 4-week intervention to assess recovery in ten stroke patients with hand motor dysfunction: one group practiced concurrent AO+MI, while the other group observed and *then* imagined the same actions. Concurrent AO+MI instructions produced larger improvements in pinch-grip strength and dexterity in the affected limb, along with more pronounced ERD in the alpha frequency band. However, given their relatively small sample more research in this area is warranted.

Three complementary studies have also demonstrated AO+MI effects on instantaneous imitation. Most recently, Bek et al. (2016) examined intentional imitation of hand movement sequences. The participants' hand movements were significantly closer to the observed action characteristics when instructed to either perform AO+MI, or pay close attention to the observed kinematics, compared to when no observation instructions were given. Since the imitation effects were equivalent across the two instruction conditions, further research is required to explore any differences in the mechanisms underlying these two observation strategies.

Eaves et al. (2012) previously demonstrated that passively observing a rhythmical distractor action produced a modest but robust automatic imitation effect in subsequently executed rhythmical actions (i.e., the participants' movement responses were biased toward the speed of the previously observed distractor). Eaves et al. (2014) then showed that this “imitation bias” was significantly stronger after participants had imagined synchronizing a rhythmical action with the distractor, regardless of the match between the MI and AO contents. This match was in terms of the rhythmical action type (e.g., imagined tooth brushing synchronized with observed window wiping) and/or dominant plane of movement. In contrast, imagining an action that conflicted with the concurrently observed action (here static MI) practically abolished the imitation bias. This provided the first empirical evidence indicating a spectrum of AO+MI states that can modulate motor execution: ranging from congruent, across coordinative to conflicting AO+MI, as first described by Vogt et al. (2013).

Eaves et al. (2016) replicated these behavioral findings, but additionally showed that the associated electrophysiological activity in mu/alpha and beta bands over the primary sensorimotor and parietal regions was significantly more pronounced in the two combined AO+MI states (that is, AO with either synchronized MI or static MI), compared to in the two single-action simulation conditions (i.e., MI and AO). These particular EEG results did not differentiate between the two AO+MI conditions, despite their contrasting behavioral effects. Synchronized AO+MI did, however, produce significantly stronger ERD in the alpha and beta bands over the rostral prefrontal cortex, compared to static AO+MI, and also compared to both AO and MI alone. This specific prefrontal involvement may reflect additional cognitive processing for aligning dual-action simulations, as discussed next.

## Conceptualizing concurrent action observation and motor imagery processes

The studies discussed above provide evidence that AO+MI is feasible and that it can significantly modulate both neurophysiological and behavioral components of motor execution. Therefore, AO and MI training should not be seen as independent interventions, but rather that their combined and simultaneous use could be more effective for practitioners (Vogt et al., 2013). Before we discuss how practitioners might incorporate AO+MI into their applied work, we first consider the need for **a theory of concurrent AO+MI processes**.

KEY CONCEPT 4 **A theory of concurrent AO+MI processes**

The existing empirical evidence can be conceptualized within a dual-action simulation account of concurrent AO+MI processes. This is an integrative and appealing theoretical approach, which can inspire novel research into AO+MI effects.

A commonly accepted framework is that both AO and MI can be regarded as two forms of motor simulation, which both involve the motor system but typically do not include motor execution (Jeannerod, 2001, 2006). It is, therefore, remarkable that these two processes have largely been studied in isolation from one another (see Vogt et al., 2013). AO is a good example of when attention is focused primarily on the somewhat unpredictable sensory inputs arising from stimuli external to the body (i.e., stimulus-orientated processing). In contrast, the content of MI does not always rely on external stimuli for its generation (i.e., stimulus-independent thought). Accordingly, AO involves a wider range of neurocognitive processes, including collaborative action (both imitative and complementary joint action), along with action prediction as the most prominent cognitive function (Springer et al., 2013). The further role of motor simulation in both the perception and conceptual processing of action (e.g., for interpreting and understanding the intentions of others) has recently come under scrutiny (e.g., Hickok, 2014; see Caramazza et al., 2014; Vannuscorps and Caramazza, 2016). Addressing this debate is beyond the scope of our current article, but it is clear that the potential impact of AO+MI instructions on this broad range of neurocognitive processes has not yet been explored. In fact, most neuroimaging studies have not controlled for the likely confound of spontaneous AO+MI occurring in paradigms that were designed to examine “pure” AO effects (Vogt et al., 2013). This is particularly worrying given that, as mentioned earlier, AO+MI can produce an increase in motor-cortical activity that is greater than the sum of the activity found during independent AO and MI states (e.g., Taube et al., 2015).

It is likely that concurrent AO+MI states are actually a common, rather than exceptional feature of daily life. Inspired by Shepard's (1984) early contribution, Vogt et al. (2013) depicted a spectrum of integrative AO+MI states existing between the two extremes: with independent AO at one end and independent MI at the other. They described how, in many daily tasks, attention needs to be flexibly biased toward one of these information sources without excluding information arriving from the other. For example, mentally rehearsing a penalty kick in soccer while watching the goalkeeper's movements, or a stroke patient who imagines their own hand movements while observing those of their clinician. From this perspective there are a range of interesting questions. Would the observed and imagined actions be represented in series (i.e., one at the expense of the other), for example, in response to switches in attentional focus? Or is it possible to co-represent two concurrent sensorimotor streams in parallel? If so, how should we envisage the relationship between two such motor representations?

The review paper by Vogt et al. (2013), along with the recent empirical evidence of Eaves et al. (2012, 2014, 2016), argues in favor of a relatively novel and integrated approach to AO+MI processes. In this account it is helpful to conceptualize the evidence for AO+MI effects using Cisek and Kalaska's (2010) framework of biased competition. This model submits that multiple sensorimotor representations are normally maintained in parallel, in the sense of action affordances. Parameters for action execution would then be selected from among the available representations. This would be achieved by different brain areas contributing their “votes” toward biasing the selection of movement parameters, in accordance with contextual information in the environment (ibid, p. 278). Within this conceptual framework it is conceivable that both an observed and an imagined action could be represented simultaneously. Presumably this would be in the sense of two concurrent and quasi-encapsulated sensorimotor streams, which could either merge or compete depending on their contents and potential usefulness for on-going action plans (Eaves et al., 2012). Thus, the relationship between these two hypothetical streams is theoretically important and can be manipulated in experiments.

Evidence showing the dissociable effects for different MI contents *during* AO was initially produced using both kinematic and electrophysiological indicators (Eaves et al., 2014, 2016). An interesting next step could now involve a more in-depth examination using multi-voxel pattern analysis (MVPA) of fMRI data into the precise anatomical substrates involved for different AO+MI states. Pilgramm et al. (2016) recently used MVPA to discriminate between different types of imagined actions purely on the basis of brain activity recorded in frontal and parietal areas, while Zabicki et al. (2016) distinguished between different action types within two modalities (imagined and executed). Furthermore, Filimon et al. (2015) also decoded the neural signatures for independent AO, MI and execution of a reaching action within brain areas jointly activated by all three modalities. Applying MVPA to fMRI data for MI of both the same and of different actions *during* AO (e.g., congruent vs. coordinative vs. conflicting AO+MI) could thus provide fresh evidence upon which to evaluate the dual-action simulation account.

A further question relates to the possible higher-order cognitive mechanisms that would preside over the interactions between dual-action representations. To this end, Eaves et al. (2016) identified pronounced electrophysiological activity in rostral prefrontal cortex specifically during synchronized AO+MI. As proposed by Burgess et al. (2005, 2007), a key role for the rostral prefrontal cortex is to route attention between information arising from sources either within the body (i.e., stimulus-independent) or the environment (i.e., stimulus-orientated), but without being involved directly in any domain-specific processing *per-se*. This “gateway hypothesis” should indeed predict increased neural activity in rostral prefrontal areas for synchronized AO+MI, because this AO+MI task requires ongoing reallocations of attention or “switching” between the externally-induced AO simulation and the internally-generated MI components.

A similar model of hierarchical control has been applied successfully in both observation (Buccino et al., 2004; Vogt et al., 2007) and imitation learning (Higuchi et al., 2012), although further empirical validations of the neurocognitive mechanism for control in dual-action simulation are now required. A limitation identified within this account, however, is that AO+MI may come at an additional cost to the user, in terms of the extra neurocognitive demands sub-serving supervisory control (Eaves et al., 2016).

## Future research opportunities

As mentioned above, a growing body of research now indicates that AO+MI can: (i) elicit increased activity in various motor regions of the brain; and (ii) influence motor behavior more directly than either AO or MI independently. Although this is a consistent finding, research into AO+MI is still in its infancy. In this section, we outline a number of unanswered questions and highlight **specific populations** that may benefit from further research into AO+MI interventions.

KEY CONCEPT 5 **Specific populations**

AO+MI interventions have the potential to improve motor function in a variety of populations. Researchers should explore the benefits of AO+MI in comparison to more traditional MI or AO interventions in sports performers, in different age groups across the lifespan and in rehabilitation.

### Motor learning

It has been claimed that AO+MI might offer optimal simulation conditions for motor learning and rehabilitation, on the basis of increased activity in motor-related brain regions during AO+MI, relative to AO or MI alone. This is clearly an attractive proposition, which should be tested empirically. A central tenet of this argument would be that greater neurophysiological activity in motor regions is beneficial for motor processes and behavioral outcomes. In contrast, Higuchi et al. (2012) presented fMRI data that indicted a trend toward increased neural efficiency (i.e., reduced activity) during both observational and, to a greater extent, physical practice. This effect was found in the regions involved in higher-order supervisory control: namely, the right motor cingulate-basal ganglia circuit and the fronto-parietal mirror circuit. It is, therefore, unclear if the increased motor-related activity induced by AO+MI training would produce changes in cortico-motor involvement that would remain beneficial throughout the various stages of motor learning. Indeed, prolonged AO+MI training may also promote cortical adaptations that differ from those in MI training (e.g., Ingram et al., 2016; see Di Rienzo et al., 2016), observational and imitation learning (see Hodges et al., 2007) and/or physical practice. Future research should investigate these effects for AO+MI within specific action categories that require different supervisory control mechanisms, such as prehensile, bimanual, and rhythmical actions, sequence learning, aiming tasks, and force production/development.

### Stroke rehabilitation

In the past two decades many researchers have highlighted the possible benefits of imagery (e.g., Sharma et al., 2006; de Vries and Mulder, 2007; Zimmermann-Schlatter et al., 2008) and observation (e.g., Holmes, 2007; Sale and Franceschini, 2012; Buccino, 2014) as effective techniques for facilitating motor recovery following stroke. This prompted an increase in research examining the effectiveness of imagery and observation as separate techniques on the recovery of motor function post-stroke. Although early research indicated that imagery may offer an effective therapy (e.g., Dijkerman et al., 2004; Page et al., 2005, 2007), results from more recent studies conflict with the early findings (e.g., Ietswaart et al., 2011; Braun et al., 2012; see Braun et al., 2013). Indeed, in Machado et al.'s (2015) meta-analysis on randomized clinical trials assessing the efficacy of imagery as a rehabilitation tool following stroke, it was concluded that imagery may not be an effective adjunct to physical therapy. Consequently, the authors suggested that further work is needed to identify the type of imagery practice best suited to stroke rehabilitation. This is particularly important given the evidence that imagery ability may be compromised following stroke (Ewan et al., 2010), potentially limiting the efficacy of such interventions.

Experiments assessing the efficacy of action observation therapy on recovery of motor function following stroke have, however, produced more consistent positive results. For example, both Ertelt et al. (2007) and Franceschini et al. (2012) demonstrated that a 4-week period of action observation therapy, involving observing activities of daily living before subsequently imitating those actions, produced improvements in both motor function and the use of the affected limb. Moreover, these benefits were retained over several months post-intervention.

In addition to contributing to the improvements in motor function, evidence from the sports domain also indicates that exposure to a video demonstration of human actions can improve aspects of imagery ability (e.g., Rymal and Ste-Marie, 2009; Wright et al., 2015). As both MI and AO may be effective in improving motor function in stroke survivors, and given the evidence that MI ability can improve following AO, combined AO+MI may prove effective in improving motor function in stroke rehabilitation. As mentioned above, there is preliminary evidence that daily AO+MI therapy over a 4-week period can increase pinch-grip strength following stroke (Sun et al., 2016), but further research to substantiate these findings would be welcome.

### Across the lifespan

Although there may be potential benefits of AO+MI in motor learning and rehabilitation, these may present differently over the lifespan. For example, action representations become less specific in older populations, which is associated with reductions in movement timing and prediction accuracy (Diersch et al., 2016). Similarly, MI ability declines in old age, particularly for more complex movement tasks, although the rate of this decline is different for temporal and spatial components of imagery ability (Kalicinski et al., 2015). AO+MI may, therefore, serve to mitigate against this loss of specificity in motor simulation, since the addition of a visual display could support and guide the degraded imagery.

In young children, MI abilities begin to emerge after the age of 5 (Molina et al., 2008), and continue to develop through adolescence and into early adulthood (Spruijt et al., 2015). In children with developmental coordination disorder (DCD), however, MI does not conform to the principles of temporal congruency observed in both healthy children and adults (Wilson et al., 2001). These children have specific impairments in generating internal representations of volitional movements; although this can be improved through MI training (Wilson et al., 2002) and, potentially, through virtual reality applications (Wilson et al., 2016). Indeed, providing concurrent AO+MI may negate the need for these individuals to allocate attentional resources to generating a visual representation of the action, allowing their efforts to be focused instead on kinesthetic imagery. Accordingly, AO+MI could be a promising therapeutic approach for this population. Consideration should, however, be given to whether the target DCD population is of an age sufficiently advanced to benefit from imagery training (c.f., Molina et al., 2008).

### Structuring the delivery of AO+MI interventions

AO+MI may offer a useful technique for facilitating motor learning and rehabilitation, although a number of important questions remain unanswered regarding how best to deliver AO+MI interventions to achieve these improvements. For example, it is currently unknown what the optimal instructions should be when delivering AO+MI interventions. According to bio-informational theory (Lang, 1977, 1979), imagery is made up of stimulus, response, and meaning propositions. Stimulus propositions refer mainly to the visual content in the image (e.g., objects and shapes in the environment), response propositions relate to feelings and responses associated with the stimuli being imagined (e.g., physiological sensations associated with movement, feelings of nervousness or arousal), and meaning propositions relate to the perceived importance and meaning attached to the imagined activity. Lang argued that imagery would be more effective if it incorporated response and meaning propositions, as opposed to only stimulus propositions.

The majority of research investigating the effect of AO+MI on neural activity has typically emphasized the inclusion of response propositions by instructing participants to engage in kinesthetic imagery, focusing on the physiological sensations involved in executing the observed movements. This decision is grounded in: (i) evidence that kinesthetic imagery activates the motor regions of the brain to a greater extent than visual imagery (e.g., Stinear et al., 2006); and (ii) the high quality visual information (provided via video demonstration) presumably negating the need to self-formulate the visual imagery component (Holmes and Calmels, 2008). While instructing kinesthetic imagery alongside action observation seems logical, research comparing different types of imagery in AO+MI is lacking. We therefore encourage researchers to compare the effects of imagery emphasizing different stimulus, response, and meaning propositions alongside action observation to identify the most effective form of imagery within AO+MI interventions.

Although the use of kinesthetic imagery instructions appears consistent in AO+MI research, there are inconsistencies across experiments in relation to the perspective used in both the action observation and imagery components of the interventions. Several studies have filmed the AO component from a first-person visual perspective (e.g., Villiger et al., 2013; Wright et al., 2014, 2016), while other studies have filmed the action from a third-person visual perspective (e.g., Eaves et al., 2014, 2016; Mouthon et al., 2015; Taube et al., 2015). In some cases, participants are instructed to explicitly image from a first person perspective, while in other cases they are only told to imagine themselves performing the observed movement, which may result in participants adopting either a first- or third-person imagery perspective, depending on their imagery perspective preference. Where there is conflict between the observation and imagery perspectives, the participant may be required to transform or rotate the video image to meet the requirements of the imagery instructions. For example, a third person video image of an action may need to be rotated and transformed into a first person imagery perspective. As cognitive tasks involving mental rotation can cause activity in motor areas of the brain (Ganis et al., 2000; Zacks, 2008; Chen et al., 2013), it is possible that the increased cortical activity commonly reported during AO+MI may reflect at least some activity resulting from transforming or rotating the observed action into a different imagery perspective, rather than functional activity related to the movement execution task. Given claims that AO+MI may offer an optimized simulation intervention for motor learning, it is important to establish the contribution that rotation and transformation of the image might make to the increased cortical activity. This could be achieved by examining cortico-motor activity during AO+MI from various imagery and observation perspective combinations. It may also be worthwhile to explore the impact of different imagery instructions, such as imagining that the observed action is a mirror image of the performer, which may remove the need to mentally rotate or transform the image.

An issue related to visual perspective is the question of whether the sense of agency is manipulated via the imagery instructions or observation video. Although AO+MI experimenters usually instruct participants to image *themselves* performing the observed movement, in most cases the agent in the video is another person. There is evidence that it may be difficult for participants to generate kinesthetic imagery when imaging from a third-person perspective, especially when the agent in the imagery is another person (Callow and Hardy, 2004). This conflict between the agent in the imagery and observation components of the intervention is problematic as it may result in less effective kinesthetic imagery, or participants switching their focus between observation of the other person performing the task and kinesthetic imagery of themselves executing the movement, rather than representing MI and AO in parallel. Future AO+MI research should therefore seek to manipulate perspective and agency within both the observation and imagery components of the intervention to identify the most appropriate method of delivering such interventions. We also encourage researchers to be clear when reporting perspective and agency issues in their methods.

Another issue is how to introduce the imagery content in the AO+MI intervention. Although it appears to be relatively easy for most healthy participants to combine the two processes, it is reasonable to assume that it may be less straightforward for individuals whose imagery ability is reduced following neurological impairment (e.g., stroke; Ewan et al., 2010; DCD, Wilson et al., 2001) or the aging process (e.g., Kalicinski et al., 2015). In such cases, one potentially beneficial method of delivering AO+MI interventions may be to introduce the imagery component of the intervention in a gradual manner. In the sport domain, Williams et al. (2013) tested a method of delivering imagery interventions called layered stimulus response training (LSRT). This process involves first reducing the mental simulation to contain only those imagery components that the participant is able to generate with ease. The complexity and realism of the image is then gradually increased over multiple practice trials by incorporating additional participant-generated stimulus, response and meaning propositions (Lang, 1977, 1979), such as sights, sounds or feelings associated with the movement task (see Cumming et al., 2016 for guidelines on LSRT). Williams et al. (2013) demonstrated that imagery interventions delivered through this method were more effective for improving golf putting performance and imagery ability in novices, compared to more traditional types of visual and motor imagery. The efficacy of LSRT is currently untested outside of the sport domain, but one avenue for research in motor learning and rehabilitation could involve establishing the effectiveness of LSRT when combined with action observation. For example, individuals could first observe a high-quality video of specific movements, rich with stimulus propositions, and be instructed to “passively” observe the video. Over multiple trials, the participant could then attempt to make the experience more realistic, by gradually incorporating additional self-selected response and/or meaning propositions, such as imaging the physiological and emotional feelings associated with performing the observed movements. Although such a layered approach to AO+MI is currently untested, given the previously discussed benefits of AO+MI and LSRT in isolation, combining the two approaches is practically appealing, particularly for those inexperienced in imagery or those who may struggle to generate imagery due to age or impairment.

## Summary and conclusion

There is now convincing evidence that concurrent AO+MI elicits increased activity in motor regions of the brain, compared to either MI or AO independently. Additionally, there is a small body of evidence indicating that combined AO+MI can also impact more directly upon motor outcomes. Thus, combined AO+MI, in conjunction with physical practice, has been recommended as a potentially more effective tool for practitioners in motor learning and rehabilitation settings. Despite the current paucity of evidence supporting this claim, the potential for important discoveries within this emerging field is rich. Novel discoveries will most likely be achieved in research adopting an integrated account of parallel AO+MI processes wherein further validations of the “dual-action” simulation approach are called for. In this context, it is important that future research establishes the best methods of delivery for AO+MI, and also which populations and tasks will benefit from this relatively novel intervention. Overall, we hope this review stimulates further research, and highlights the potential for AO+MI to enhance the work of applied practitioners who seek to improve motor abilities.

## Author contributions

DE, MR, PH, and DW all contributed to the planning of this review paper. DE, MR, and DW all wrote sections of the article and provided feedback on draft versions of the manuscript. PH provided critical comments and amendments to draft versions of the manuscript.

### Conflict of interest statement

The authors declare that the research was conducted in the absence of any commercial or financial relationships that could be construed as a potential conflict of interest.
